# A defucosylated anti-CD44 monoclonal antibody 5-mG_2a_-f exerts antitumor effects in mouse xenograft models of oral squamous cell carcinoma

**DOI:** 10.3892/or.2020.7735

**Published:** 2020-08-14

**Authors:** Junko Takei, Mika K. Kaneko, Tomokazu Ohishi, Hideki Hosono, Takuro Nakamura, Miyuki Yanaka, Masato Sano, Teizo Asano, Yusuke Sayama, Manabu Kawada, Hiroyuki Harada, Yukinari Kato

**Affiliations:** 1 Department of Antibody Drug Development, Tohoku University Graduate School of Medicine, Aoba-ku, Sendai, Miyagi 980-8575, Japan; 2 Department of Oral and Maxillofacial Surgery, Graduate School of Medical and Dental Sciences, Tokyo Medical and Dental University, Bunkyo-ku, Tokyo 113-8510, Japan; 3 Institute of Microbial Chemistry (BIKAKEN), Numazu, Microbial Chemistry Research Foundation, Numazu-shi, Shizuoka 410-0301, Japan; 4 New Industry Creation Hatchery Center, Tohoku University, Sendai, Miyagi 980-8575, Japan

**Keywords:** CD44, monoclonal antibody, antibody-dependent cellular cytotoxicity, complement-dependent cytotoxicity, antitumor activity, oral cancer

## Abstract

CD44 is widely expressed on the surface of most tissues and all hematopoietic cells, and regulates many genes associated with cell adhesion, migration, proliferation, differentiation, and survival. CD44 has also been studied as a therapeutic target in several cancers. Previously, an anti-CD44 monoclonal antibody (mAb), C_44_Mab-5 (IgG_1_, kappa) was established by immunizing mice with CD44-overexpressing Chinese hamster ovary (CHO)-K1 cells. C_44_Mab-5 recognized all CD44 isoforms, and showed high sensitivity for flow cytometry and immunohistochemical analysis in oral cancers. However, as the IgG_1_ subclass of C_44_Mab-5 lacks antibody-dependent cellular cytotoxicity (ADCC) and complement-dependent cytotoxicity (CDC), the antitumor activity of C_44_Mab-5 could not be determined. In the present study, we converted the mouse IgG_1_ subclass antibody C_44_Mab-5 into an IgG_2a_ subclass antibody, 5-mG_2a_, and further produced a defucosylated version, 5-mG_2a_-f, using FUT8-deficient ExpiCHO-S (BINDS-09) cells. Defucosylation of 5-mG_2a_-f was confirmed using fucose-binding lectins, such as AAL and PhoSL. The dissociation constants (*K*_D_) for 5-mG_2a_-f against SAS and HSC-2 oral cancer cells were determined through flow cytometry to be 2.8×10^−10^ M and 2.6×10^−9^ M, respectively, indicating that 5-mG_2a_-f possesses extremely high binding affinity. Furthermore, immunohistochemical staining using 5-mG_2a_-f specifically stained the membranes of oral cancer cells. *In vitro* analysis demonstrated that 5-mG_2a_-f showed moderate ADCC and CDC activities against SAS and HSC-2 oral cancer cells. *In vivo* analysis revealed that 5-mG_2a_-f significantly reduced tumor development in SAS and HSC-2 ×enografts in comparison to control mouse IgG, even after injection seven days post-tumor inoculation. Collectively, these results suggest that treatment with 5-mG_2a_-f may represent a useful therapy for patients with CD44-expressing oral cancers.

## Introduction

Oral cancers account for about 2% of all cancer cases diagnosed worldwide (1). More than 350,000 individuals are diagnosed with oral cancer every year, and oral cancers prove fatal for approximately 170,000 of these people. Major risk factors for oral cancer include the use of alcohol and tobacco (2). Although decreased drinking and smoking have resulted in a decline in the incidence of oral cancer, recent studies have reported an increase in the number of young patients diagnosed with these diseases (3,4).

CD44 is known to be expressed in many cell types, including epithelial cells, fibroblasts, endothelial cells, and leukocytes (5). CD44 plays important roles in cell proliferation, adhesion, and migration (6). The CD44 gene consists of 20 exons (7). The smallest isoform is the standard form of CD44 (CD44s), which possesses 10 exons; other possible isoforms are categorized as CD44 variants (CD44v), which are generated by alternatively spliced transcripts (8). Post-translational modifications such as *N*- and *O*-glycosylation and heparan sulfate modification also augment the diversity of CD44 (9,10). Both CD44s and CD44v are overexpressed in many cancers; however, a pattern of expression remains to be elucidated.

One of the CD44 variants, CD44v6, was first identified as contributing to cancer metastasis, and CD44v6-specific monoclonal antibodies (mAbs) were found to inhibit metastasis of rat pancreatic cancers (11,12). Some CD44v6 isoforms act as co-receptors for receptor tyrosine kinases (RTKs) such as MET and vascular endothelial growth factor receptor (VEGFR)-2 (13–15). The transfection of CD44v4-7 cDNA confers a metastatic phenotype in non-metastatic cells (16). Another CD44 variant, CD44v3, binds to several heparan sulfate-binding growth factors such as fibroblast growth factors (FGFs) and heparin-binding epidermal growth factor (HB-EGF), and induces tumor progression (17,18). Several CD44 variants were also reported as prognostic markers in head and neck, lung, colorectal, breast, and hepatocellular cancers (19–23).

Many mAbs have been developed to target CD44 (24–26). mAbs that neutralize contact between hyaluronic acid and CD44 have been shown to inhibit anchorage-independent growth of murine mammary carcinoma cells and human colon carcinoma cells (24). Anti-CD44 mAbs were also found to exhibit significant antitumor activity in mouse xenograft models of human cancers (25,26). Previously, we established clone C_44_Mab-5 (IgG_1_, kappa) using Cell-Based Immunization and Screening (CBIS) (27). C_44_Mab-5 recognized both CD44s and CD44v isoforms, and demonstrated high sensitivity for flow cytometry and immunohistochemical analysis in oral cancers. Because the IgG_1_ subclass of C_44_Mab-5 lacks antibody-dependent cellular cytotoxicity (ADCC) and complement-dependent cytotoxicity (CDC), antitumor activity of C_44_Mab-5 could not be determined.

In this study, we converted the IgG_1_ subclass C_44_Mab-5 into a mouse IgG_2a_ subclass mAb, 5-mG_2a_, and further produced a defucosylated version, 5-mG_2a_-f, using FUT8-deficient ExpiCHO-S cells (28). We then investigated whether 5-mG_2a_-f exhibited ADCC, CDC and antitumor activities against oral cancers.

## Materials and methods

### 

#### Cell lines

Oral squamous carcinoma cell lines including HSC-2 (oral cavity) and SAS (tongue) were obtained from the Japanese Collection of Research Bioresources Cell Bank (JCRB; Osaka, Japan). Chinese hamster ovary (CHO)-K1 was obtained from the American Type Culture Collection (ATCC). CD44v3-10 plus N-terminal PA16 tag-overexpressed CHO-K1 (CHO/PA16-CD44v3-10) was generated by transfection of pCAG/PA16-CD44v3-10 to CHO-K1 cells using the Neon Transfection System (Thermo Fisher Scientific, Inc.). The PA16 tag consists of 16 amino acids (GLEGGVAMPGAEDDVV) (27). HSC-2 and SAS cells were cultured in Dulbecco's modified Eagle's medium (DMEM; Nacalai Tesque, Inc.), and CHO-K1 and CHO/PA16-CD44v3-10 were cultured in RPMI-1640 medium (Nacalai Tesque, Inc.), supplemented with 10% heat-inactivated fetal bovine serum (FBS; Thermo Fisher Scientific Inc.), 100 units/ml of penicillin, 100 µg/ml streptomycin, and 0.25 µg/ml amphotericin B (Nacalai Tesque, Inc.) at 37°C in a humidified atmosphere containing 5% CO_2_.

#### Antibodies

Mouse anti-CD44s mAb C_44_Mab-5 (IgG_1_, kappa) was developed as previously described (27). Mouse IgG was purchased from Sigma-Aldrich Corp. (Merck KGaA). To generate recombinant C_44_Mab-5 (recC_44_Mab-5), cDNAs of C_44_Mab-5 heavy and light chains were subcloned into pCAG-Neo and pCAG-Ble vectors (FUJIFILM Wako Pure Chemical Corporation), respectively. To generate 5-mG_2a_-f, appropriate V_H_ cDNA of mouse C_44_Mab-5 and C_H_ of mouse IgG_2a_ were subcloned into pCAG-Neo vector, and light chain of C_44_Mab-5 was subcloned into pCAG-Ble vector. Vectors were transfected into ExpiCHO-S or BINDS-09 (FUT8-deficient ExpiCHO-S cells) using the ExpiCHO Expression System (28). recC_44_Mab-5 and 5-mG_2a_-f were purified using Protein G-Sepharose (GE Healthcare Bio-Sciences).

#### Animals

All animal experiments were performed in accordance with relevant guidelines (e.g. ARRIVE guidelines) and regulations (e.g. 3R regulations) to minimize animal suffering and distress in the laboratory (29,30). Seventy female BALB/c nude mice (6 weeks old, 15–18 g) were purchased from Charles River (Kanagawa, Japan). Animal studies for ADCC and antitumor activity were approved by the Institutional Committee for Experiments of the Institute of Microbial Chemistry (permit number: 2020-003). Mice were maintained in a pathogen-free environment (23±2°C, 55±5% humidity) on 11 h light/13 h dark cycle with food and water supplied *ad libitum* during the experimental period. Mice were monitored for health and weight every 1 or 5 days. Experiment duration was three weeks. A bodyweight loss exceeding 25% and a maximum tumor size exceeding 3,000 mm^3^ were identified as humane endpoints. Mice were euthanized by cervical dislocation, and the death was verified by respiratory arrest and cardiac arrest.

#### Enzyme-linked immunosorbent assay (ELISA)

C_44_Mab-5 and 5-mG_2a_-f were immobilized on Nunc Maxisorp 96-well immunoplates (Thermo Fisher Scientific Inc.) at 1 µg/ml for 30 min. After blocking using SuperBlock buffer (Thermo Fisher Scientific Inc.) containing 0.5 mM CaCl_2_, the plates were incubated with biotin-labeled lectins, such as *Aleuria aurantia* lectin (AAL; Vector Laboratories), *Pholiota squarrosa* lectin (PhoSL; J-OIL MILLS, Inc.) (31), and concanavalin A (ConA; Vector Laboratories), followed by 1:3,000 diluted peroxidase-conjugated streptavidin (Agilent Technologies). The enzymatic reaction was produced using a 1-Step Ultra TMB-ELISA (Thermo Fisher Scientific Inc.). The optical density was measured at 655 nm using an iMark microplate reader (Bio-Rad Laboratories, Inc.).

#### Flow cytometry

Cells were harvested by brief exposure to 0.25% trypsin/1 mM ethylenediaminetetraacetic acid (EDTA; Nacalai Tesque, Inc.). After washing with 0.1% bovine serum albumin (BSA) in phosphate-buffered saline (PBS), cells were treated with primary mAbs for 30 min at 4°C and subsequently with Alexa Fluor 488-conjugated anti-mouse IgG (1:1,000; Cell Signaling Technology, Inc.). Fluorescence microscopy data were collected using an EC800 Cell Analyzer (Sony Corp.).

#### Immunohistochemical analyses

Histologic sections (4-µm thick) of an oral cancer tissue microarray (catalogue number: OR481; US Biomax Inc.) were directly autoclaved in citrate buffer (pH 6.0; Agilent Technologies Inc.) for 20 min. Sections were then incubated with 1 µg/ml primary mAbs for 1 h at room temperature and treated using an Envision+ Kit (Agilent Technologies) for 30 min. Color was developed using 3,3′-diaminobenzidine tetrahydrochloride (DAB; Agilent Technologies Inc.) for 2 min, and sections were then counterstained with hematoxylin (FUJIFILM Wako Pure Chemical Corporation). Hematoxylin and eosin (H&E) staining (FUJIFILM Wako Pure Chemical Corporation) was performed using consecutive tissue sections. Leica DMD108 (Leica Microsystems GmbH) was used to examine the sections and obtain images.

#### Determination of the binding affinity

Cells were suspended in 100 µl of serially diluted mAbs (0.3 ng/ml-5 µg/ml), followed by the addition of Alexa Fluor 488-conjugated anti-mouse IgG (1:200; Cell Signaling Technology, Inc.). Fluorescence microscopy data were collected using an EC800 Cell Analyzer (Sony Corp.). The dissociation constant (*K*_D_) was calculated by fitting binding isotherms to built-in one-site binding models in GraphPad PRISM 8 (GraphPad Software, Inc.).

#### Western blot analysis

Cell lysates (10 µg) were boiled in sodium dodecyl sulfate (SDS) sample buffer (Nacalai Tesque, Inc.). Proteins were separated on 5–20% polyacrylamide gels (FUJIFILM Wako Pure Chemical Corporation) and transferred onto polyvinylidene difluoride (PVDF) membranes (Merck KGaA). After blocking with 4% skim milk (Nacalai Tesque, Inc.) in PBS with 0.05% Tween 20, the membranes were incubated with 10 µg/ml of an anti-CD44 mAb [clone C_44_Mab-46 (mouse IgG_1_, kappa)]; available from Antibody Bank of Tohoku University (ABTU; Miyagi, Japan); http://www.med-tohoku-antibody.com/topics/001_paper_antibody_PDIS.htm#antiCD44) or 1 µg/ml of anti-β-actin (clone AC-15; cat. no. A5441; Sigma-Aldrich Corp.; Merck KGaA). This was followed by incubation with peroxidase-conjugated anti-mouse immunoglobulins (Agilent Technologies Inc.). Finally, protein bands were detected with ImmunoStar LD (FUJIFILM Wako Pure Chemical Corporation) using a Sayaca-Imager (DRC Co., Ltd.).

#### Reverse transcription-polymerase chain reaction (RT-PCR)

Total RNAs were prepared from cell lines using an RNeasy Mini Prep Kit (Qiagen Inc.). The initial cDNA strand was synthesized using SuperScript IV Reverse Transcriptase (Thermo Fisher Scientific, Inc.) by priming nine random oligomers and an oligo(dT) primer according to the manufacturer's instructions. We performed 35 cycles of PCR for amplification using HotStarTaq DNA Polymerase (Qiagen Inc.) with 0.2 µM of primer sets: Human CD44 sense (5′-GAAAGGAGCAGCACTTCAGG-3′), human CD44 antisense (5′-ACTGCAATGCAAACTGCAAGC-3′), GAPDH sense (5′-CAATGACCCCTTCATTGACC-3′), and GAPDH antisense (5′-GTCTTCTGGGTGGCAGTGAT-3′).

#### ADCC

Six six-week-old female BALB/c nude mice were purchased from Charles River (Kanagawa, Japan). After euthanization by cervical dislocation, spleens were removed aseptically and single-cell suspensions obtained by forcing spleen tissues through a sterile cell strainer (352360, BD Falcon, Corning, Inc.) using a syringe. Erythrocytes were lysed with a 10-sec exposure to ice-cold distilled water. Splenocytes were washed with DMEM and resuspended in DMEM with 10% FBS and used as effector cells. Target cells were labeled with 10-µg/ml Calcein AM (Thermo Fisher Scientific, Inc.) and resuspended in the same medium. The target cells (2×10^4^ cells/well) were plated in 96-well plates and mixed with effector cells, anti-CD44s antibodies, or control IgG (mouse IgG_2a_) (Sigma-Aldrich Corp.; Merck KGaA). After a 5-h incubation, the Calcein AM release of supernatant from each well was measured. Fluorescence intensity was determined using a microplate reader (Power Scan HT) (BioTek Instruments) with an excitation wavelength of 485 nm and an emission wavelength of 538 nm. Cytolytic activity (as % of lysis) was calculated using the equation: % lysis=(E-S)/(M-S) ×100, where E is the fluorescence of the combined target and effector cells, S is the spontaneous fluorescence of the target cells only, and M is the maximum fluorescence measured after lysing all cells with a buffer containing 0.5% Triton X-100, 10 mM Tris-HCl (pH 7.4), and 10 mM of EDTA.

#### CDC

Cells in DMEM supplemented with 10% FBS were plated in 96-well plates (2×10^4^ cells/well), and incubated for 5 h at 37°C with either anti-CD44s antibodies or control IgG (mouse IgG_2a_) (Sigma-Aldrich Corp.; Merck KGaA) and 10% rabbit complement (Low-Tox-M Rabbit Complement; Cedarlane Laboratories). To assess cell viability, an MTS [3-(4,5-dimethylthiazol-2-yl)-5-(3-carboxymethoxyphenyl)-2-(4-sulfophenyl)-2H-tetrazolium; inner salt] assay was performed using a CellTiter 96^®^AQueous assay kit (Promega Corp.).

#### 3D cell proliferation assay

3D cell proliferation was measured with the CellTiter-Glo^®^ 3D cell viability assay (Promega Corp.) according to the manufacturer's instructions. Briefly, the cells were plated (2,000 cells/100 µl/well) in triplicate in 96-well ultra low attachment plates (Corning Inc.) with PBS or 100 µg/ml of mouse IgG_2a_ and an anti-CD44 mAb (5-mG_2a_-f) in DMEM containing 10% FBS. The cell viability was measured after 48 h of incubation. The CellTiter-Glo^®^ 3D reagent was added into wells in a 1:1 dilution (100 µl volume in well:100 µl of reagent) and then the plates were shaken for 5 min on an orbital shaker and incubated at room temperature for an additional 25 min. The luminescent signal was read using an EnSpire multi-plate reader (Perkin Elmer). Images were taken using an Evolution MP camera (Media Cybernetics). The proliferation rate was calculated relative to the control (PBS was added instead of the antibodies).

#### Antitumor activity of 5-mG_2a_-f in the xenografts of oral cancers

Sixty-four six-week-old female BALB/c nude mice were purchased from Charles River (Kanagawa, Japan) and used at 10 weeks of age. HSC-2 and SAS cells (0.3 ml of 1.33×10^8^ cells/ml in DMEM) were mixed with 0.5 ml BD Matrigel Matrix Growth Factor Reduced (BD Biosciences). One hundred microliters of this suspension (5×10^6^ cells) was injected subcutaneously into the left flank. After day 1 (protocol-1) or day 7 (protocol-2), 100 µg of 5-mG_2a_-f and control mouse IgG (Sigma-Aldrich Corp.; Merck KGaA) in 100 µl PBS were injected intraperitoneally (i.p.) into treated and control mice, respectively. Additional antibodies were then injected on days 7 and 14 (protocol-1) or on days 14 and 21 (protocol-2). Nineteen days (protocol-1) or 27 days (protocol-2) after cell implantation, all mice were euthanized by cervical dislocation and tumor diameters and volumes were determined as previously described (32).

#### Statistical analyses

All data are expressed as mean ± standard error of the mean (SEM). Statistical analysis was carried out using ANOVA following Tukey-Kramer's test for ADCC and CDC. Sidak's multiple comparisons test was used for tumor volume and mouse weight, or Welch's t test for tumor weight and 3D cell proliferation assay using GraphPad Prism 7 (GraphPad Software, Inc.). P<0.05 was adopted as a level of statistical significance.

## Results

### 

#### Production and characterization of 5-mG_2a_-f, a core-fucose-deficient mouse IgG_2a_-type anti-CD44 antibody

**Figure 1.**
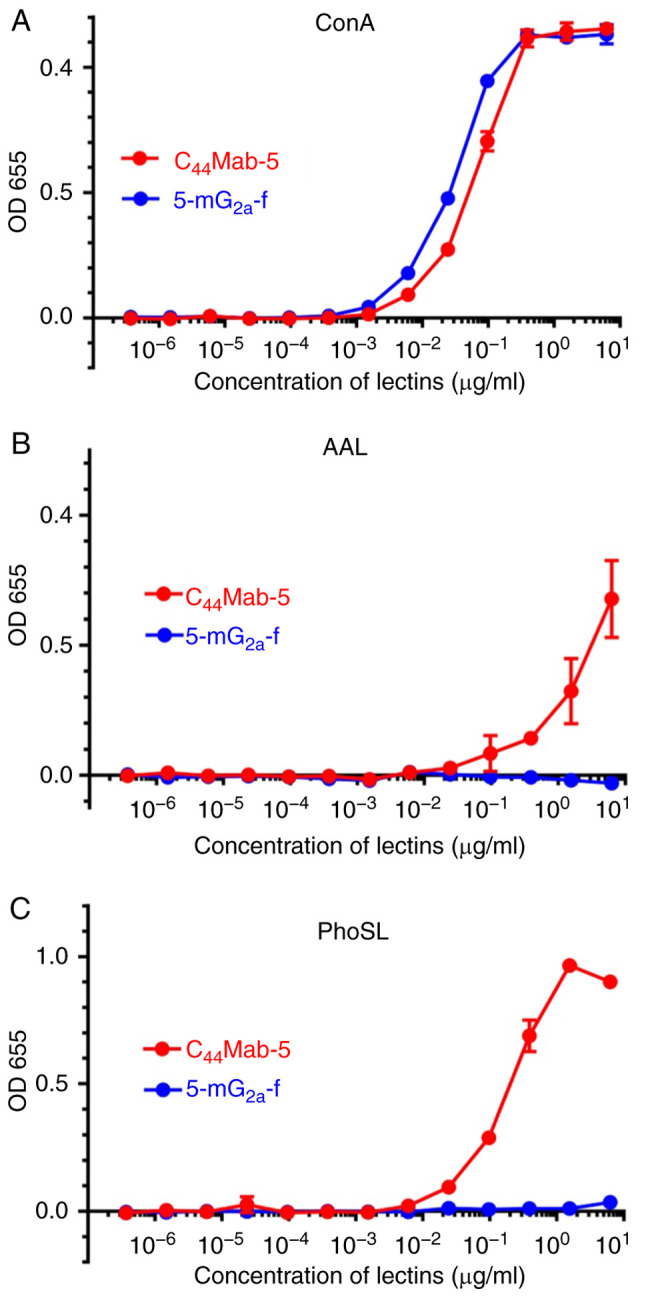
Confirmation of defucosylation of 5-mG_2a_-f by enzyme-linked immunosorbent assay (ELISA) using lectins. (A) C_44_Mab-5 and 5-mG_2a_-f were immobilized and incubated with biotin-labeled concanavalin A (Con A), followed by peroxidase-conjugated streptavidin. The enzymatic reaction was produced using a 1-Step Ultra TMB-ELISA. (B) C_44_Mab-5 and 5-mG_2a_-f were immobilized and incubated with biotin-labeled *Aleuria aurantia* lectin (AAL), followed by peroxidase-conjugated streptavidin. The enzymatic reaction was produced using a 1-Step Ultra TMB-ELISA. (C) C_44_Mab-5 and 5-mG_2a_-f were immobilized and incubated with biotin-labeled *Pholiota squarrosa* lectin (PhoSL), followed by peroxidase-conjugated streptavidin. The enzymatic reaction was produced using a 1-Step Ultra TMB-ELISA.

As mouse IgG_2a_ possesses high ADCC and CDC activities (33), we first produced a mouse IgG_2a_ version of mouse IgG_1_ C_44_Mab-5 by subcloning appropriate V_H_ cDNA of C_44_Mab-5 and C_H_ of mouse IgG_2a_ into pCAG-Neo vector, and light chain of C_44_Mab-5 into pCAG-Ble vector. This IgG_2a_-type of C_44_Mab-5 is henceforth referred to as 5-mG_2a_. We additionally produced a core-fucose-deficient type of 5-mG_2a_, henceforth referred to as 5-mG_2a_-f, using the BINDS-09 cell line (FUT8-knockout Expi-CHO-S cell line) (28). Defucosylation of 5-mG_2a_-f was confirmed using lectins such as *Aleuria aurantia* lectin (AAL, fucose binder) (34) and *Pholiota squarrosa* lectin (PhoSL, core fucose binder) (31). Concanavalin A (ConA, mannose binder) (35) was used as a control. Both C_44_Mab-5 and 5-mG_2a_-f were detected using ConA (Fig. 1A). C_44_Mab-5, but not 5-mG_2a_-f, was detected using AAL (Fig. 1B) or PhoSL (Fig. 1C), indicating that 5-mG_2a_-f was defucosylated.

**Figure 2.**
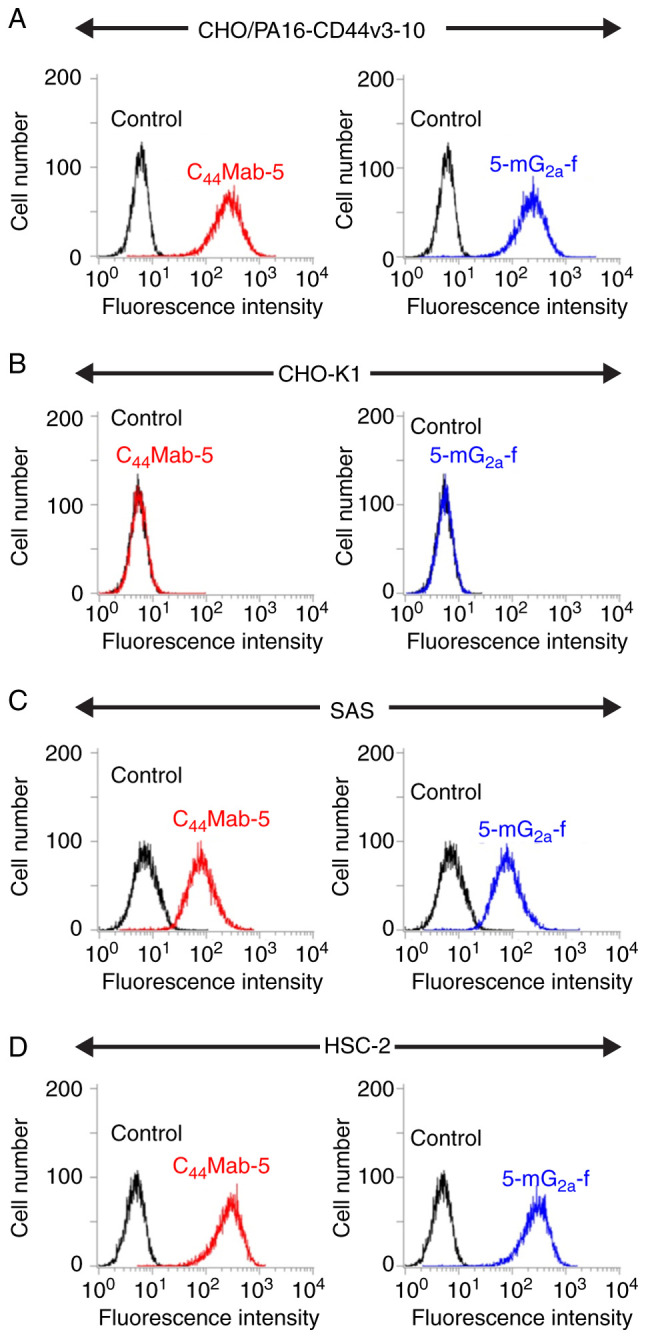
Flow cytometry using anti-CD44 mAbs. (A) CHO/PA16-CD44v3-10 cells were treated with C_44_Mab-5 and 5-mG_2a_-f (1 µg/ml), followed by secondary antibodies. (B) CHO-K1 cells were treated with C_44_Mab-5 and 5-mG_2a_-f (1 µg/ml), followed by secondary antibodies. (C) SAS cells were treated with C_44_Mab-5 and 5-mG_2a_-f (1 µg/ml), followed by secondary antibodies. (D) HSC-2 cells were treated with C_44_Mab-5 and 5-mG_2a_-f (1 µg/ml), followed by secondary antibodies. The black line represents the negative control. mAbs, monoclonal antibodies.

We examined the sensitivity of 5-mG_2a_-f in CHO cells expressing CD44v3-10 plus N-terminal PA16 tag (CHO/PA16-CD44v3-10) and in oral squamous cell carcinoma (OSCC) cell lines (SAS and HSC-2) using flow cytometry. Both C_44_Mab-5 and 5-mG_2a_-f reacted with CHO/PA16-CD44v3-10 cells (Fig. 2A), but not with CHO-K1 cells (Fig. 2B). Both C_44_Mab-5 and 5-mG_2a_-f reacted with SAS cells (Fig. 2C) and HSC-2 cells (Fig. 2D), indicating that both mAbs showed high sensitivity against SAS and HSC-2 cells.

As shown in Fig. S1A, CD44 was not detected by an anti-CD44s mAb (C_44_Mab-46) in both SAS and HSC-2 cells presumably because the CD44 expression level in those cells might be low for the detection in western blot analysis. Then, we performed RT-PCR analysis for detection of CD44. As shown in Fig. S1B, the multiple bands of CD44v were detected in SAS and HSC-2 cells using PCR, indicating that CD44v is expressed in SAS and HSC-2 cells.

**Figure 3.**
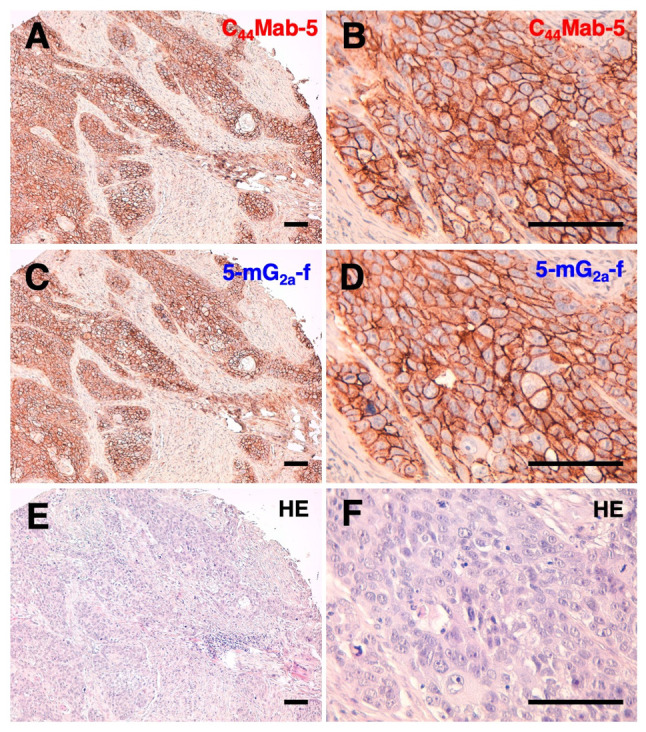
Immunohistochemical analysis using anti-CD44 mAbs against oral squamous cell carcinomas (OSCCs). (A and B) Consecutive tissue sections of OSCC were incubated with 1 µg/ml of C_44_Mab-5 for 1 h at room temperature followed by treatment with an Envision+ kit for 30 min. Color was developed using DAB for 2 min, and sections were then counterstained with hematoxylin. (C and D) Consecutive tissue sections of OSCC were incubated with 1 µg/ml of 5-mG_2a_-f for 1 h at room temperature followed by treatment with an Envision+ kit for 30 min. Color was developed using DAB for 2 min, and sections were then counterstained with hematoxylin. (E and F) Hematoxylin and eosin (HE) staining of consecutive tissue sections of OSCC. Scale bar, 100 µm. mAbs, monoclonal antibodies.

Next, we performed immunohistochemical analysis on oral cancer cell lines. Representative images are shown in Fig. 3. Both C_44_Mab-5 (Fig. 3A and B) and 5-mG_2a_-f (Fig. 3C and D) stained the plasma membrane of oral cancer cells. The sensitivity of 5-mG_2a_-f was similar with that of C_44_Mab-5. Both C_44_Mab-5 and 5-mG_2a_-f stained 33/38 cases (86.8%) of OSCCs of the tissue microarray. Hematoxylin & eosin (H&E) staining of consecutive tissue sections of OSCC is shown in Fig. 3E and F.

**Figure 4.**
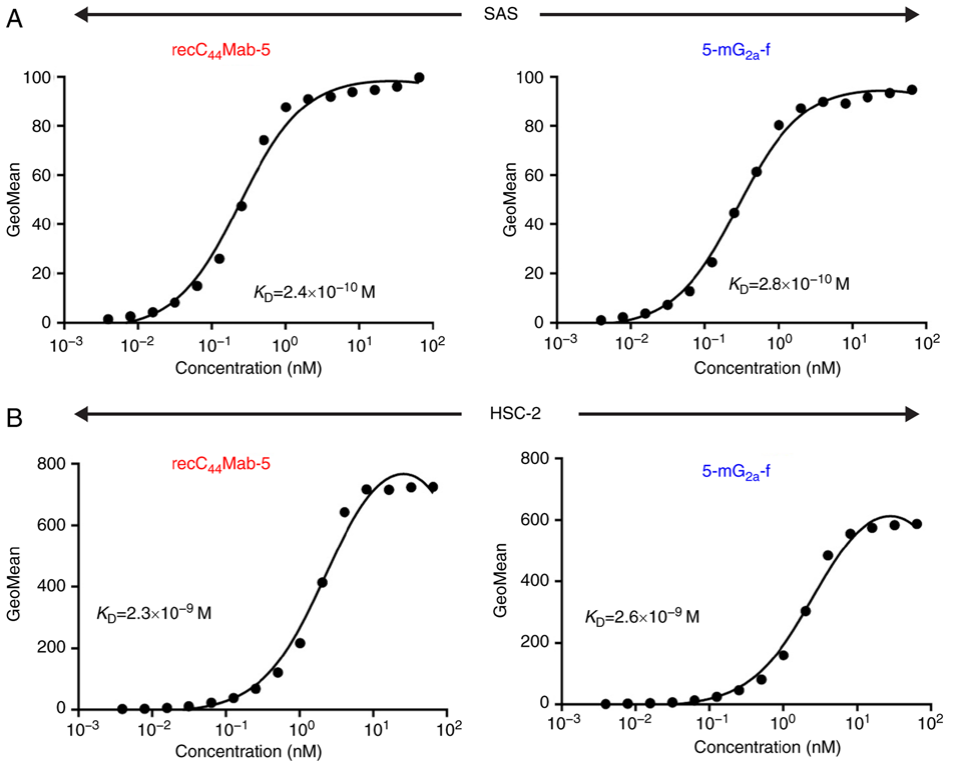
Determination of the binding affinity of anti-CD44 mAbs for oral cancer cells using flow cytometry. (A) SAS cells were suspended in 100 µl of serially diluted mAbs (0.3 ng/ml-5 µg/ml), followed by the addition of Alexa Fluor 488-conjugated anti-mouse IgG. Fluorescence data were collected using an EC800 Cell Analyzer. (B) HSC-2 cells were suspended in 100 µl of serially diluted mAbs (0.3 ng/ml-5 µg/ml), followed by the addition of Alexa Fluor 488-conjugated anti-mouse IgG. Fluorescence data were collected using an EC800 Cell Analyzer. *K*_D_, dissociation constants; mAbs, monoclonal antibodies.

We performed a kinetic analysis of the interactions of recC_44_Mab-5 and 5-mG_2a_-f with SAS and HSC-2 oral cancer cell lines using flow cytometry. As shown in Fig. 4A, the dissociation constant (*K*_D_) for recC_44_Mab-5 in SAS cells was 2.4×10^−10^ M. In contrast, the *K*_D_ for 5-mG_2a_-f in SAS cells was 2.8×10^−10^ M (Fig. 4A). The binding affinity of 5-mG_2a_-f in SAS cells was very similar to that of recC_44_Mab-5. Likewise, the *K*_D_ for recC_44_Mab-5 against HSC-2 was 2.3×10^−9^ M (Fig. 4B). In contrast, the *K*_D_ for 5-mG_2a_-f in HSC-2 cells was 2.6×10^−9^ M (Fig. 4B). The binding affinity of 5-mG_2a_-f in HSC-2 cells was very similar to that of recC_44_Mab-5. The binding affinity of 5-mG_2a_-f in SAS was 9.3-fold higher than that against HSC-2.

#### ADCC and CDC activities of 5-mG_2a_-f in oral cancer cell lines

**Figure 5.**
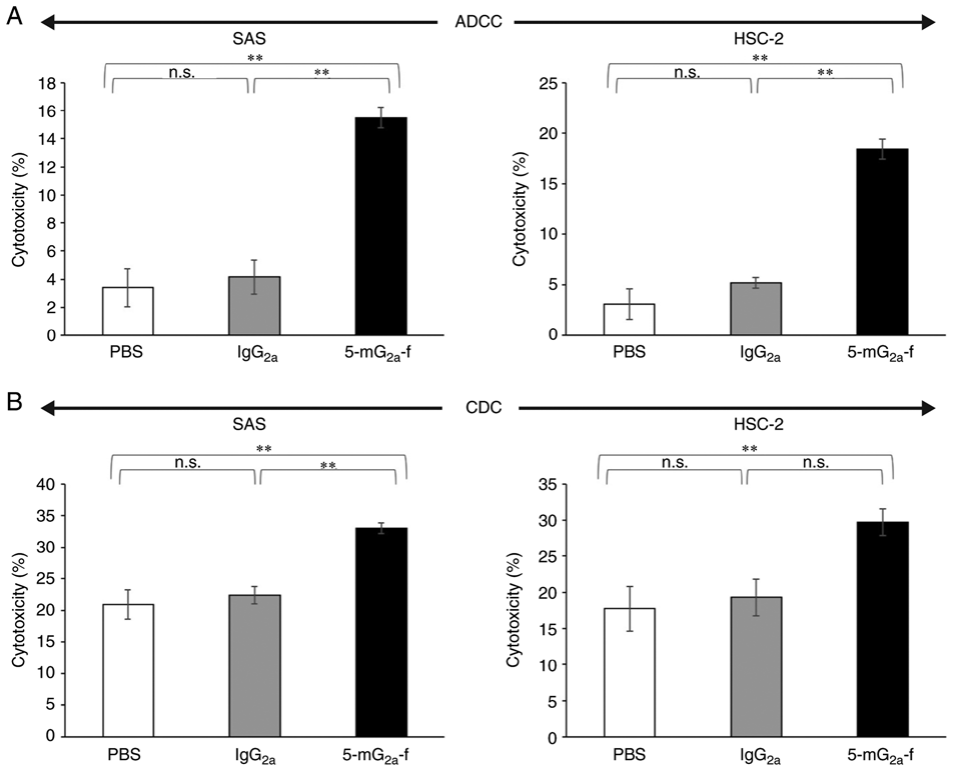
Evaluation of ADCC and CDC activities by 5-mG_2a_-f. (A) ADCC activities by 5-mG_2a_-f, control mouse IgG_2a_, and control PBS in SAS and HSC-2 cells. (B) CDC activities by 5-mG_2a_-f, control mouse IgG_2a_, and control PBS in SAS and HSC-2 cells. Values are mean ± SEM. Asterisk indicates statistical significance (**P<0.01; n.s., not significant; ANOVA and Tukey-Kramer's test). ADCC, antibody-dependent cellular cytotoxicity; CDC, complement-dependent cytotoxicity.

Because the mouse IgG_1_ subclass of C_44_Mab-5 does not possess ADCC or CDC activities, we synthesized a mouse IgG_2a_ subclass mAb, and further defucosylated it to augment those activities. In this study, we examined whether the developed 5-mG_2a_-f induced ADCC and CDC in CD44-expressing oral cancer cell lines, such as SAS and HSC-2 cells. As shown in Fig. 5A, 5-mG_2a_-f exhibited higher ADCC (16% cytotoxicity) in SAS cells compared with that of control PBS (3.4% cytotoxicity; P<0.01) and control mouse IgG_2a_ treatment (4.2% cytotoxicity; P<0.01). Similarly, 5-mG_2a_-f exhibited higher ADCC (18% cytotoxicity) against HSC-2 cells compared with that of control PBS (3.1% cytotoxicity; P<0.01) and control mouse IgG_2a_ treatment (5.2% cytotoxicity; P<0.01), indicating that ADCC in SAS cells is similar with that in HSC-2 cells, despite the binding affinity of 5-mG_2a_-f in SAS cells being 9.3-fold higher than in HSC-2 cells (Fig. 4). As shown in Fig. 5B, 5-mG_2a_-f exhibited slightly higher CDC (33% cytotoxicity) in SAS cells compared with control PBS (21% cytotoxicity; P<0.01) and control mouse IgG_2a_ treatment (22% cytotoxicity; P<0.01). Similarly, 5-mG_2a_-f exhibited slightly higher CDC (30% cytotoxicity) in HSC-2 cells compared with control PBS (18% cytotoxicity; P<0.01) and control mouse IgG_2a_ treatment (19% cytotoxicity; not significant). Although ADCC/CDC activities of 5-mG_2a_-f in oral cancer cells are not outstanding, 5-mG_2a_-f may exert antitumor activity against oral cancer cells *in vivo*.

#### The influence of 5-mG_2a_-f in oral cancer cell lines in anchorage-independent condition

Next, we investigated whether 5-mG_2a_-f inhibits cell growth of SAS and HSC-2 cells in anchorage-independent condition. As shown in Fig. S2A, both SAS and HSC-2 cells grew in anchorage-independent condition for 48 h. In contrast, an anti-CD44 mAb (5-mG_2a_-f) did not inhibit the growth of SAS or HSC-2 compared to control mouse IgG_2a_ (Fig. S2B), indicating that 5-mG_2a_-f did not affect the cell growth of oral cancer cell lines in anchorage-independent condition.

#### Antitumor activities of 5-mG_2a_-f in the mouse xenografts of SAS oral cancer cells

**Figure 6.**
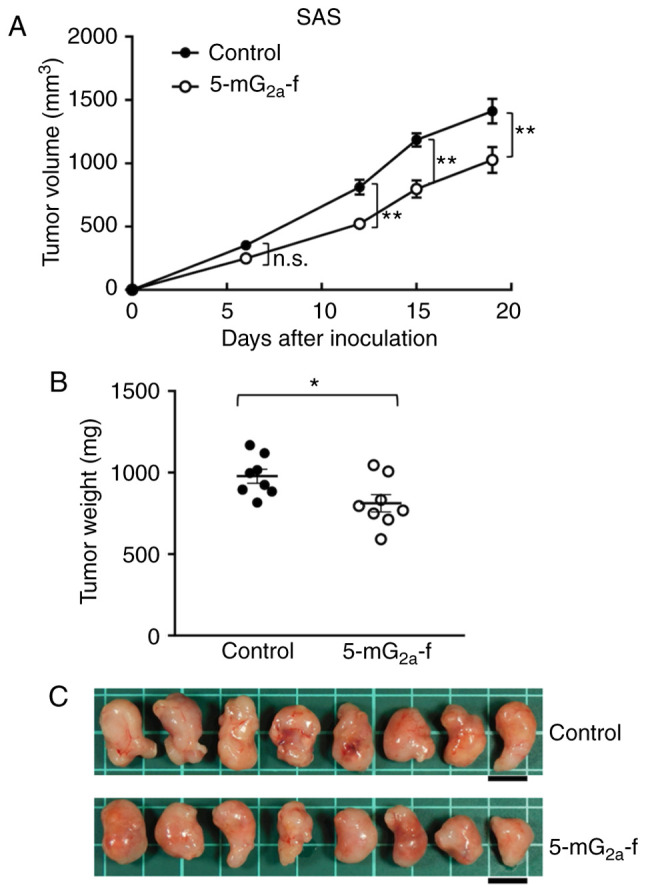
Evaluation of antitumor activity of 5-mG_2a_-f (from day 1) in SAS xenografts. (A) SAS cells (5×10^6^ cells) were injected subcutaneously into the left flank. After day 1, 100 µg of 5-mG_2a_-f and control mouse IgG in 100 µl PBS were injected i.p. into treated and control mice, respectively. Additional antibodies were then injected on days 7 and 14. Tumor volume was measured on days 6, 12, 15, and 19. Values are mean ± SEM. Asterisk indicates statistical significance (**P<0.01; n.s., not significant; ANOVA and Sidak's multiple comparisons test). (B) Tumors of SAS xenografts were resected from 5-mG_2a_-f and control mouse IgG groups. Tumor weight on day 19 was measured from excised xenografts. Values are mean ± SEM. Asterisk indicates statistical significance (*P<0.05, Welch's t test). (C) Resected tumors of SAS xenografts from 5-mG_2a_-f and control mouse IgG groups on day 19. Scale bar, 1 cm.

SAS cells were subcutaneously implanted into the flanks of nude mice. In protocol-1, 5-mG_2a_-f (100 µg) and control mouse IgG (100 µg) were injected i.p. three times into the mice, on days 1, 7, and 14 after SAS cell injections. Tumor volume was measured on days 6, 12, 15, and 19. Tumor development was significantly reduced in the 5-mG_2a_-f-treated mice on days 12, 15, and 19 in comparison to the IgG-treated control mice (Fig. 6A). Tumor volume on day 19 was reduced by 27% after 5-mG_2a_-f treatment. Tumors from 5-mG_2a_-f-treated mice weighed significantly less than tumors from IgG-treated control mice (16.9% reduction, Fig. 6B). Resected tumors on day 19 are depicted in Fig. 6C. Control and 5-mG_2a_-f-treated SAS xenograft mice are shown on day 19 in Fig. S3A and B, respectively. Total body weights did not significantly differ between the two groups (Fig. S3C).

**Figure 7.**
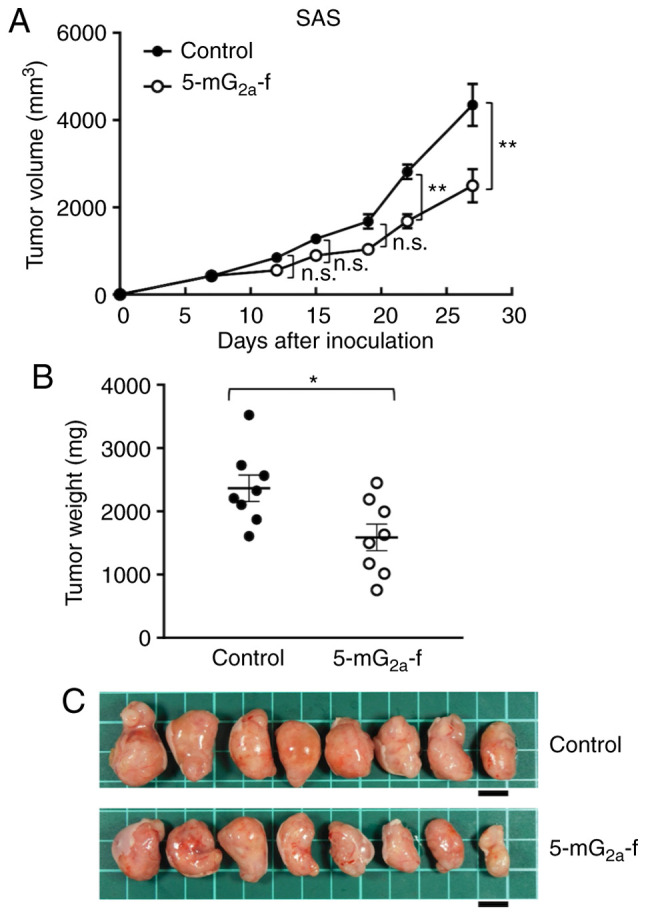
Evaluation of antitumor activity of 5-mG_2a_-f (from day 7) in SAS xenografts. (A) SAS cells (5×10^6^ cells) were injected subcutaneously into the left flank. After day 7, 100 µg of 5-mG_2a_-f and control mouse IgG in 100 µl PBS were injected i.p. into treated and control mice, respectively. Additional antibodies were then injected on days 14 and 21. Tumor volume was measured on days 7, 12, 15, 19, 22, and 27. Values are mean ± SEM. Asterisk indicates statistical significance (**P<0.01; n.s., not significant; ANOVA and Sidak's multiple comparisons test). (B) Tumors of SAS xenografts were resected from 5-mG_2a_-f and control mouse IgG groups. Tumor weight on day 27 was measured from excised xenografts. Values are mean ± SEM. Asterisk indicates statistical significance (*P<0.05, Welch's t test). (C) Resected tumors of SAS xenografts from 5-mG_2a_-f and control mouse IgG groups on day 27. Scale bar, 1 cm.

In protocol-2 of the SAS xenograft models, tumor formation of 16 SAS-bearing mice was observed on day 7. Then, these 16 SAS-bearing mice were divided into a 5-mG_2a_-f-treated group and a control group. On days 7, 14, and 21 after SAS cell injections into the mice, 5-mG_2a_-f (100 µg) and control mouse IgG (100 µg) were injected i.p. into the mice. Tumor formation was observed in mice in both treated and control groups. Tumor volume was measured on days 7, 12, 15, 19, 22, and 27. 5-mG_2a_-f-treated mice displayed significantly reduced tumor development on days 22 and 27 in comparison to IgG-treated control mice (Fig. 7A). Tumor volume reduction by 5-mG_2a_-f was 43% on day 27. Tumors from the 5-mG_2a_-f-treated mice weighed significantly less than tumors from the IgG-treated control mice (27.1% reduction, Fig. 7B). Resected tumors on day 27 are depicted in Fig. 7C. Control and 5-mG_2a_-f-treated SAS xenograft mice are shown on day 27 in Fig. S4A and B, respectively. Total body weights did not significantly differ between the two groups (Fig. S4C). These results indicate that 5-mG_2a_-f reduced the growth of SAS xenografts effectively, even when 5-mG_2a_-f was injected 7 days post-SAS cell injections in mice.

#### Antitumor activities of 5-mG_2a_-f in mouse xenografts of HSC-2 oral cancer cells

**Figure 8.**
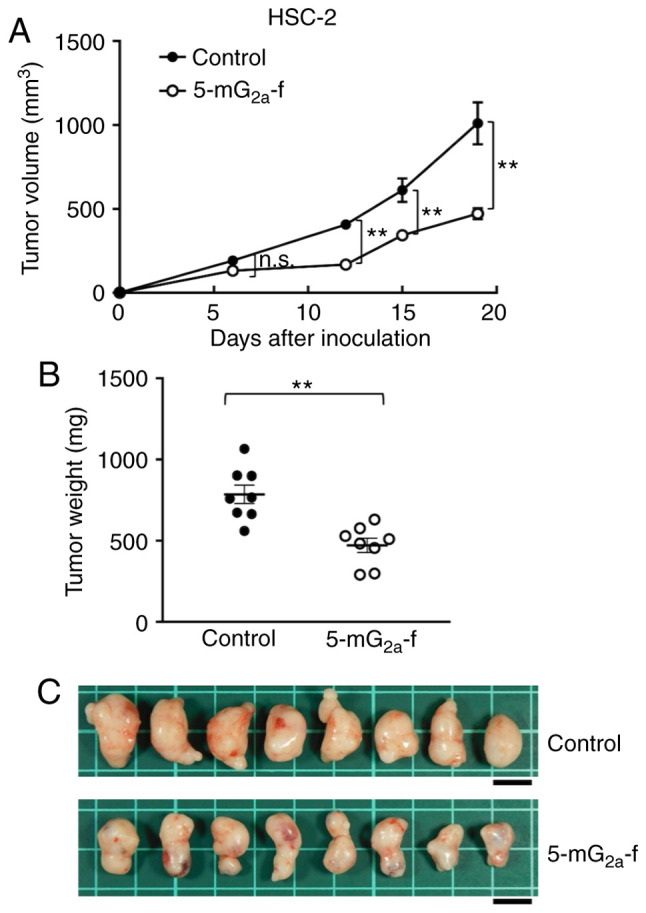
Evaluation of antitumor activity of 5-mG_2a_-f (from day 1) in HSC-2 ×enografts. (A) HSC-2 cells (5×10^6^ cells) were injected subcutaneously into the left flank. After day 1, 100 µg of 5-mG_2a_-f and control mouse IgG in 100 µl PBS were injected i.p. into treated and control mice, respectively. Additional antibodies were then injected on days 7 and 14. Tumor volume was measured on days 6, 12, 15, and 19. Values are mean ± SEM. Asterisk indicates statistical significance (**P<0.01; n.s., not significant, ANOVA and Sidak's multiple comparisons test). (B) Tumors of HSC-2 ×enografts were resected from 5-mG_2a_-f and control mouse IgG groups. Tumor weight on day 19 was measured from excised xenografts. Values are mean ± SEM. Asterisk indicates statistical significance (**P<0.01, Welch's t test). (C) Resected tumors of HSC-2 ×enografts from 5-mG_2a_-f and control mouse IgG groups on day 19. Scale bar, 1 cm.

In a second xenograft model of oral cancers, HSC-2 cells were subcutaneously implanted into the flanks of nude mice. In protocol-1 of HSC-2 ×enograft models, 5-mG_2a_-f (100 µg) and control mouse IgG (100 µg) were injected i.p. three times into the mice, on days 1, 7, and 14 after HSC-2 cell injections into the mice. Tumor volume was measured on days 6, 12, 15, and 19. 5-mG_2a_-f-treated mice displayed significantly reduced tumor development on days 12, 15, and 19 in comparison to IgG-treated control mice (Fig. 8A). Tumor volume reduction by 5-mG_2a_-f was 53% on day 19. Tumors from 5-mG_2a_-f-treated mice weighed significantly less than HSC-2 tumors from IgG-treated control mice (44.1% reduction, Fig. 8B). Resected tumors on day 19 are depicted in Fig. 8C. Control and 5-mG_2a_-f-treated HSC-2 ×enograft mice are shown on day 19 in Fig. S5A and B, respectively. Total body weights did not significantly differ between the two groups (Fig. S5C).

**Figure 9.**
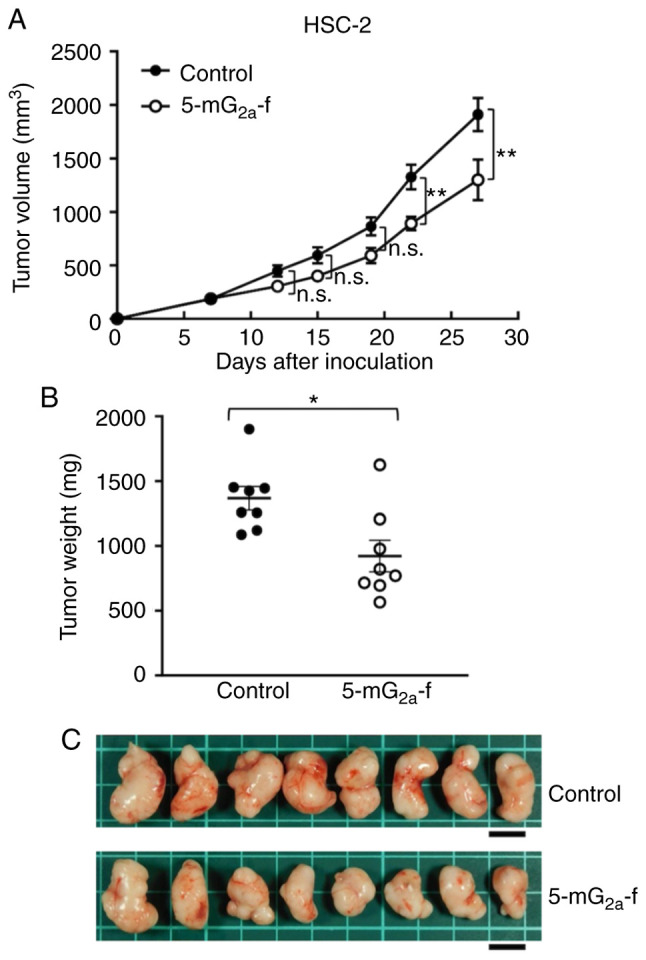
Evaluation of antitumor activity of 5-mG_2a_-f (from day 7) in HSC-2 ×enografts. (A) HSC-2 cells (5×10^6^ cells) were injected subcutaneously into the left flank. After day 7, 100 µg of 5-mG_2a_-f and control mouse IgG in 100 µl PBS were injected i.p. into treated and control mice, respectively. Additional antibodies were then injected on days 14 and 21. Tumor volume was measured on days 7, 12, 15, 19, 22, and 27. Values are mean ± SEM. Asterisk indicates statistical significance (**P<0.01; n.s., not significant; ANOVA and Sidak's multiple comparisons test). (B) Tumors of HSC-2 ×enografts were resected from 5-mG_2a_-f and control mouse IgG groups. Tumor weight on day 27 was measured from excised xenografts. Values are mean ± SEM. Asterisk indicates statistical significance (*P<0.05, Welch's t test). (C) Resected tumors of HSC-2 ×enografts from 5-mG_2a_-f and control mouse IgG groups on day 27. Scale bar, 1 cm.

In protocol-2 of the HSC-2 ×enograft models, tumor formation of 16 HSC-2-bearing mice was observed on day 7. Then, these 16 HSC-2-bearing mice were divided into a 5-mG_2a_-f-treated group and a control group. On days 7, 14, and 21 after cell injections into the mice, 5-mG_2a_-f (100 µg) and control mouse IgG (100 µg) were injected i.p. into the mice. Tumor volume was measured on days 7, 12, 15, 19, 22, and 27. 5-mG_2a_-f-treated mice displayed significantly reduced tumor development on days 22 and 27 in comparison to IgG-treated control mice (Fig. 9A). Tumor volume reduction in 5-mG_2a_-treated mice was 32% on day 27. Tumors from 5-mG_2a_-f-treated mice weighed significantly less than tumors from IgG-treated control mice (27.1% reduction, Fig. 9B). Resected tumors on day 27 are depicted in Fig. 9C. Control and 5-mG_2a_-f-treated HSC-2 ×enograft mice are shown on day 27 in Fig. S6A and B, respectively. Total body weights did not significantly differ between the two groups (Fig. S6C). These results indicate that 5-mG_2a_-f reduced the growth of HSC-2 ×enografts effectively, even when 5-mG_2a_-f was injected 7 days post-HSC-2 cell injections in mice.

## Discussion

In the present study, we investigated whether anti-CD44 mAbs are advantageous for the treatment of oral cancers. We previously developed a sensitive and specific anti-CD44 mAb, C_44_Mab-5, but were unable to demonstrate antitumor activity because the IgG_1_ subclass does not possess ADCC/CDC activities (27). In this study, we developed this antibody into an IgG_2a_ subclass antibody, and augmented ADCC activity using a defucosylated variant. Oral cancers comprise several histological tumor types, such as squamous cell carcinoma (SCC), adenocarcinoma, mucoepidermoid carcinoma, and adenoid cystic carcinoma. Among these, SCC accounts for over 90% of all oral cancers (36). Therefore, we used SAS and HSC-2 cell lines, which are derived from SCC, and investigated ADCC/CDC and antitumor activities.

The most effective treatment of OSCC depends upon its clinical stage. Stage-I and -II (early stages) are treated via surgery or radiotherapy alone. In contrast, stage-III and -IV (advanced stages) require a combination of surgery, radiotherapy, and chemotherapy (37). For chemotherapy of OSCCs, cisplatin is mainly used, and is often combined with docetaxel and 5-fluorouracil (38,39). Other anticancer agents, such as paclitaxel, carboplatin, and methotrexate can be also used for OSCCs (40), but effective molecular targeting drugs, such as antibody therapies, are limited.

Recently, cetuximab, a mouse-human chimeric mAb (IgG_1_) that targets epidermal growth factor receptor (EGFR), was approved by the Food and Drug Administration (FDA) in the USA for treatment of oral cancers (41). Cetuximab has been shown effective against locoregionally advanced head and neck cancer and recurrent or metastatic squamous cell carcinoma of the head and neck (41–43). Although advances in diagnosis and therapeutic techniques have improved the overall 5-year survival rate to 70%, the 5-year survival rate in stage IV is only 40%; therefore, further treatments must be developed (44). In our recent study, we also developed a sensitive and specific mAb (EMab-17) against EGFR, and demonstrated its ADCC/CDC and antitumor activity against SAS and HSC-2 ×enografts (32). Although we showed that EMab-17 could potentially be used for antibody-based therapy for EGFR-expressing OSCC, the difference between cetuximab and EMab-17 has not been clarified. Several studies characterizing EMab-17, including epitope mapping and signal induction in OSCC cells, are currently ongoing.

In another recent study, HER2 was shown to be expressed in oral cancers, and an anti-HER2 mAb (H_2_Mab-19) demonstrated antitumor activity (45). Therefore, anti-HER2 therapies using trastuzumab could be effective for the treatment of oral cancers. HER2 expression was reported in only 1.4% of immunohistochemical analyses of oral cancer (46), although it is expressed in 10.4% of breast cancers (47). Therefore, targeting only HER2 may be insufficient for conquering oral cancers. As antitumor effects of combined gefitinib and trastuzumab or cetuximab and trastuzumab treatment on head and neck SCC (HNSCC) were demonstrated *in vitro* (48,49), those few oral cancer patients displaying HER2 overexpression/amplification may possibly benefit from anti-HER2 therapy.

Furthermore, we previously investigated whether podocalyxin (PODXL) may be a therapeutic target in OSCC using anti-PODXL mAbs (50). We engineered an anti-PODXL mAb of IgG_1_ subclass (PcMab-47) into a mouse IgG_2a_-type mAb (47-mG_2a_) to increase ADCC. We further developed 47-mG2a-f, a core fucose-deficient variant of 47-mG_2a_ to further augment its ADCC. *In vivo* analysis revealed that 47-mG_2a_-f, but not 47-mG_2a_, exhibited antitumor activity in SAS and HSC-2 ×enograft models at a dose of 100 µg/mouse/week administered three times. Although both 47-mG_2a_ and 47-mG_2a_-f exhibited antitumor activity in HSC-2 ×enograft models at a dose of 500 µg/mouse/week administered twice, 47-mG_2a_-f also demonstrated higher antitumor activity than 47-mG_2a_, indicating that a core fucose-deficient anti-PODXL mAb could be useful for antibody-based therapy against PODXL-expressing OSCCs. Therefore, we used the core-fucose-deficient anti-CD44 mAb (5-mG_2a_-f) for treating CD44-expressing oral cancers.

In this study, we demonstrated that 5-mG_2a_-f exerts ADCC/CDC activities *in vitro*, and antitumor activities *in vivo*. Importantly, 5-mG_2a_-f effectively reduced the growth of SAS and HSC-2 ×enografts, even when 5-mG_2a_-f was injected 7 days after cell implantations into the mice. However, tumor volume reduction of SAS and HSC-2 on day 27 by 5-mG_2a_-f was still only 43 and 32%, respectively, indicating that anti-CD44 therapy might not be robust enough for conquering most oral cancers. One potential reason for this weak antitumor activity is the lower ADCC activity and CDC activity of 5-mG_2a_-f, despite high binding activity in SAS cells (*K*_D_: 2.8×10^−10^ M) and HSC-2 cells (*K*_D_: 2.6×10^−9^ M).

In our previous report, C_44_Mab-5 detected CD44s (27). Although the binding epitope of C_44_Mab-5 may potentially be located between exon-1 and exon-5, we have not been able to determine the exact binding epitope of C_44_Mab-5, likely because C_44_Mab-5 recognizes the tertiary structure of CD44 rather simple peptides or glycans. Because the binding epitope is critical for ADCC/CDC activities of mAbs, other anti-CD44 mAbs of various epitopes will need to be developed in future studies.

Targeting multiple targets, such as EGFR, HER2, PODXL, and CD44 may be needed for effective therapy to conquer oral cancers. Another important goal is the targeting of cancer-specific antigens using a cancer-specific mAb (CasMab) because EGFR, HER2, PODXL, and CD44 are widely expressed in normal tissues. We previously established CasMab against podoplanin (PDPN), which is expressed in many cancers, including oral cancers (51–54). In xenograft models with HSC-2 cells, a mouse-human chimeric mAb, chLpMab-23, exerted antitumor activity using human natural killer cells, indicating that chLpMab-23 may be useful for antibody therapy against PDPN-expressing oral cancers (54). In the future study, cancer-specific anti-CD44 mAbs may also be developed that can reduce the adverse effects of traditional antibody therapy.

## Supplementary Material

Supporting Data

## Data Availability

The datasets used and/or analyzed during the study are available from the corresponding author on reasonable request.

## Abbreviations

AAL: *Aleuria aurantia* lectin
ABTU: Antibody Bank of Tohoku University
ADCC: antibody-dependent cellular cytotoxicity
ATCC: American Type Culture Collection
BSA: bovine serum albumin
CasMab: cancer-specific mAb
CBIS: cell-based immunization and screening
CDC: complement-dependent cytotoxicity
CD44s: CD44 standard
CD44v: CD44 variant
CHO: Chinese hamster ovary
Con A: concanavalin A
DMEM: Dulbecco's modified Eagle's medium
EDTA: ethylenediaminetetraacetic acid
ELISA: enzyme-linked immunosorbent assay
FBS: fetal bovine serum
FDA: Food and Drug Administration
FGF: fibroblast growth factor
HNSCC: head and neck squamous cell carcinoma
HB-EGF: heparin-binding epidermal growth factor
JCRB: Japanese Collection of Research Bioresources Cell Bank
mAb: monoclonal antibody
OSCC: oral squamous cell carcinoma
PBS: phosphate-buffered saline
PDPN: podoplanin
PhoSL: *Pholiota squarrosa* lectin
PODXL: podocalyxin
PVDF: polyvinylidene difluoride
RTKs: receptor tyrosine kinases
RT-PCR: reverse transcription-polymerase chain reaction
SCC: squamous cell carcinoma
SDS: sodium dodecyl sulfate
SEM: standard error of the mean
VEGFR: vascular endothelial growth factor receptor
