# Supplementary material for: A defucosylated anti-CD44 monoclonal antibody 5-mG2a-f exerts antitumor effects in mouse xenograft models of oral squamous cell carcinoma
Source: Oncol Rep. 2020 Aug 14;44(5):1949–60. doi: 10.3892/or.2020.7735 (PMC7550977; doi:10.3892/or.2020.7735)

Figure S1. Expression analysis of CD44s and CD44v in SAS and HSC-2 cells. (A) Western blot analysis using an anti-CD44 mAb. The cell lysates of CHO/CD44s, CHO/CD44v3-v10, CHO-K1, SAS, and HSC-2 cells were subjected to SDS-PAGE and transferred onto the PVDF membranes. The membranes were incubated with C_{44}Mab-46 and anti-b-actin (AC-15), followed by secondary antibodies. (B) RT-PCR analysis. We performed 35 cycles of PCR for amplification using HotStarTaq DNA Polymerase with primer sets of CD44 and GAPDH. mAb, monoclonal antibody; RT-PCR, reverse transcription-polymerase chain reaction.

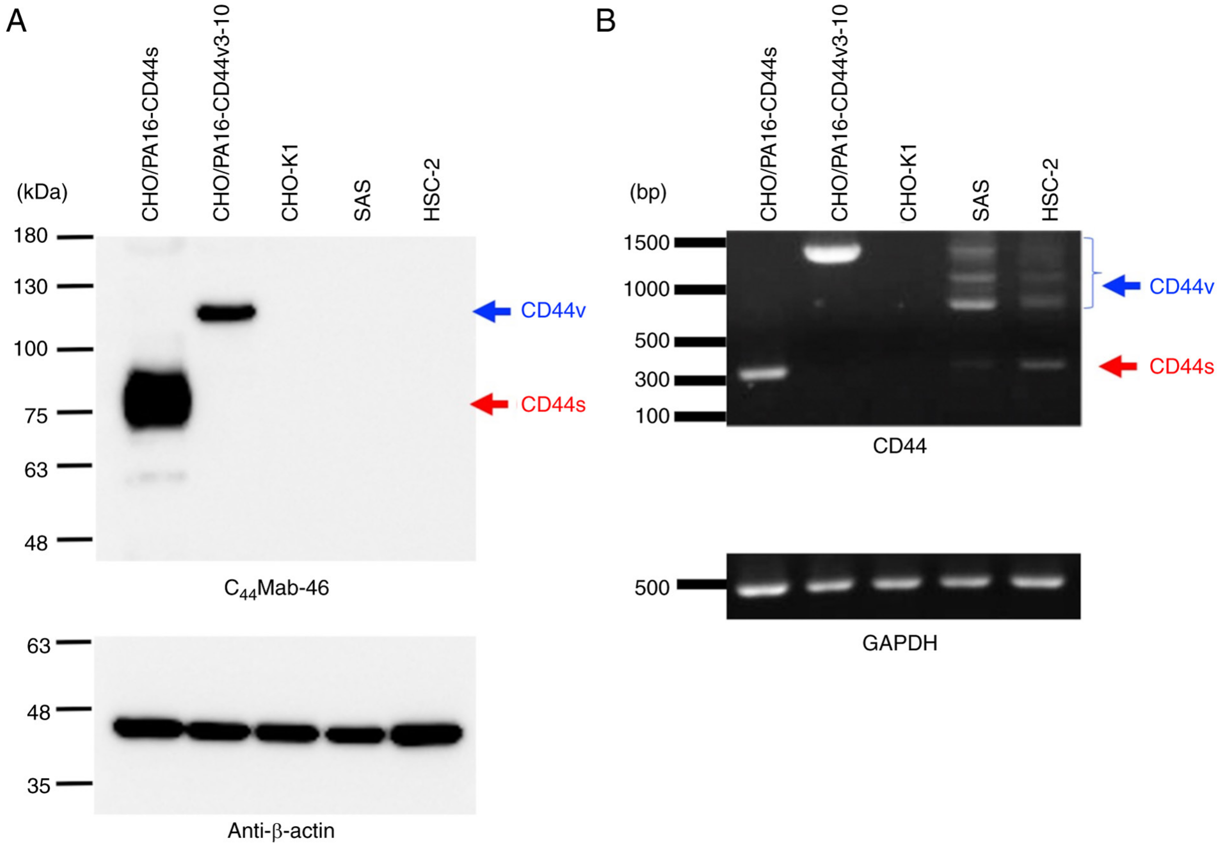

Figure S2. Proliferation of SAS and HSC-2 cells in 3D cultures and the influence of an anti-CD44 antibody (5-mG2a-f) on the 3D cell proliferation. (A) Optical microscopic images of SAS and HSC-2 cells after 48 h incubation. Scale bar, 200 mm. (B) SAS and HSC-2 cells were treated with control (PBS), mouse IgG_{2a}, and an anti-CD44 antibody (5-mG_{2a}-f) for 48 h. The 3D cell proliferation rate of mouse IgG_{2a} or 5-mG_{2a}-f treatment was calculated relative to the control. The values are means ± SEM. n.s., not significant (Welch's t test).

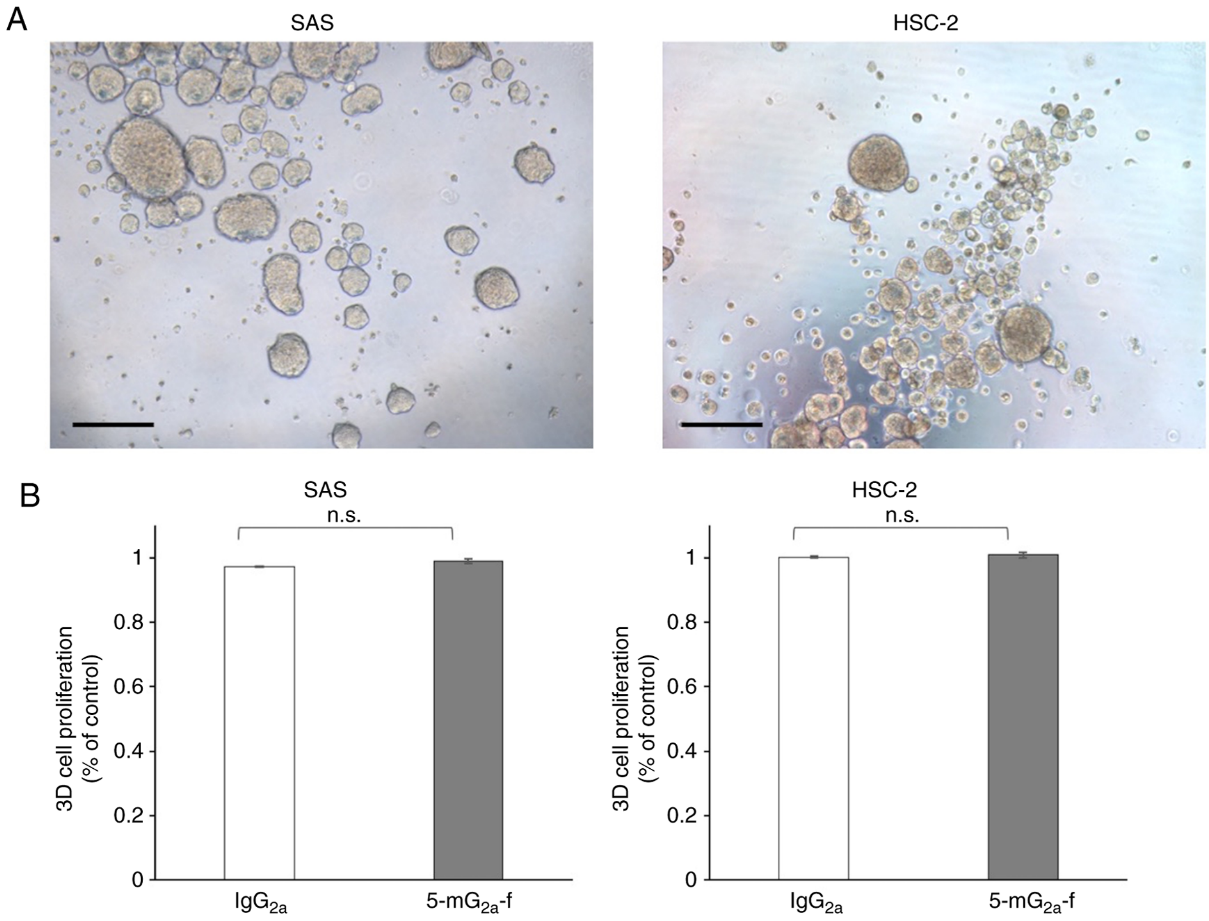

Figure S3. Body weights of mice implanted with SAS xenografts and injected with 5-mG_{2a}-f after day 1. SAS cells ($5x10^6$ cells) were injected subcutaneously into the left flank. After day 1, 100 μg of 5-mG_{2a}-f and control mouse IgG in 100 μl PBS were injected i.p. into treated and control mice, respectively. Additional antibodies were then injected on days 7 and 14. (A) Body appearance of mice with the SAS xenografts of the control group on day 19. (B) Body appearance of mice with the SAS xenografts of the 5-mG_{2a}-f group on day 19. (C) Body weights of the mice with the SAS xenografts of 5-mG_{2a}-f group and control group were recorded on days 5, 7, 12, 15, and 19. Values are mean ± SEM. n.s., not significant (ANOVA and Sidak's multiple comparisons test).

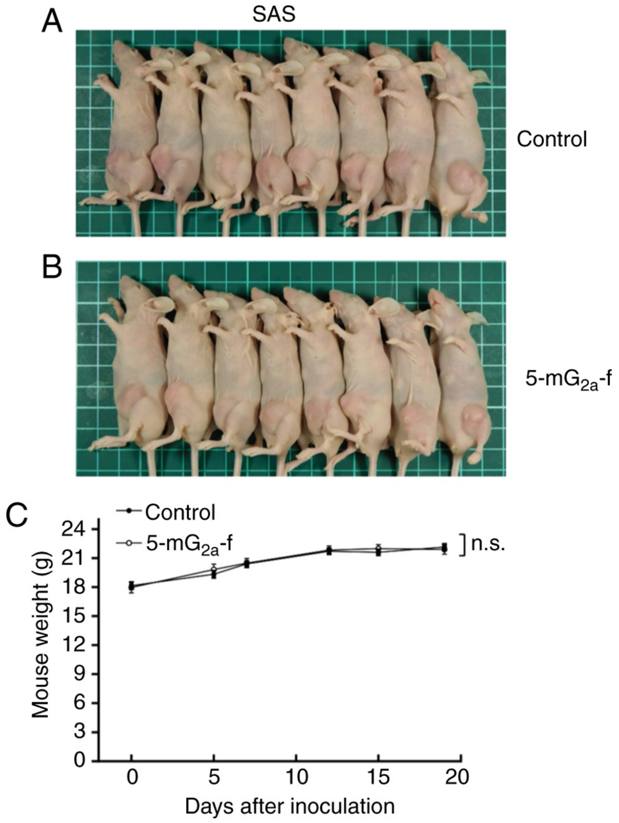

Figure S4. Body weights of mice implanted with SAS xenografts and injected with 5-mG$_{2a}$-f after day 7. SAS cells (5x10^6 cells) were injected subcutaneously into the left flank. After day 7, 100 μg of 5-mG$_{2a}$-f and control mouse IgG in 100 μl PBS were injected i.p. into treated and control mice, respectively. Additional antibodies were then injected on days 14 and 21. (A) Body appearance of mice with the SAS xenografts of the control group on day 27. (B) Body appearance of mice with the SAS xenografts of the 5-mG$_{2a}$-f group on day 27. (C) Body weights of the mice with the SAS xenografts of 5-mG$_{2a}$-f group and control group were recorded on days 7, 12, 15, 19, 22, 26, and 27. Values are mean ± SEM. n.s., not significant (ANOVA and Sidak's multiple comparisons test).

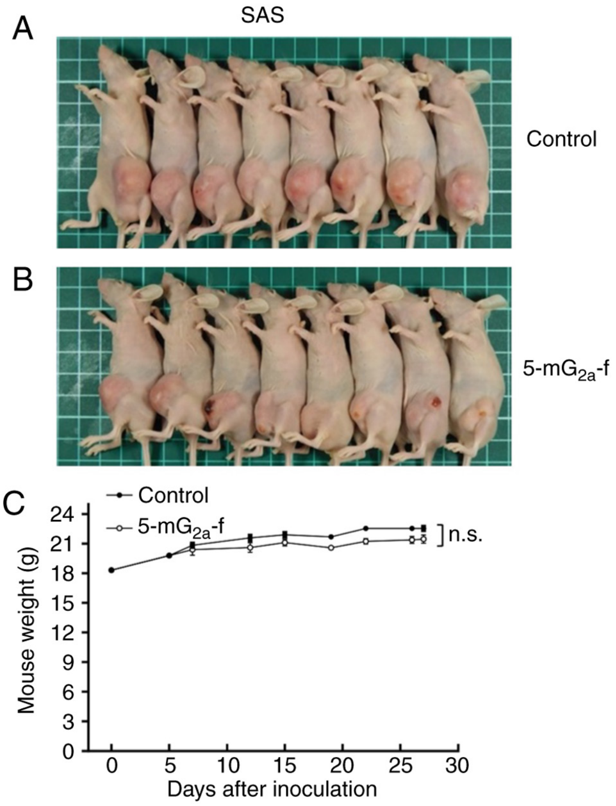

Figure S5. Body weights of mice implanted with HSC-2 xenografts and injected with 5-mG_{2a}-f after day 1. HSC-2 cells ($5x10^6$ cells) were injected subcutaneously into the left flank. After day 1, 100 μg of 5-mG_{2a}-f and control mouse IgG in 100 μl PBS were injected i.p. into treated and control mice, respectively. Additional antibodies were then injected on days 7 and 14. (A) Body appearance of mice with the HSC-2 xenografts of the control group on day 19. (B) Body appearance of mice with the HSC-2 xenografts of the 5-mG_{2a}-f group on day 19. (C) Body weights of the mice with the HSC-2 xenografts of 5-mG_{2a}-f group and control group were recorded on days 5, 7, 12, 15, and 19. Values are mean ± SEM. n.s., not significant (ANOVA and Sidak's multiple comparisons test).

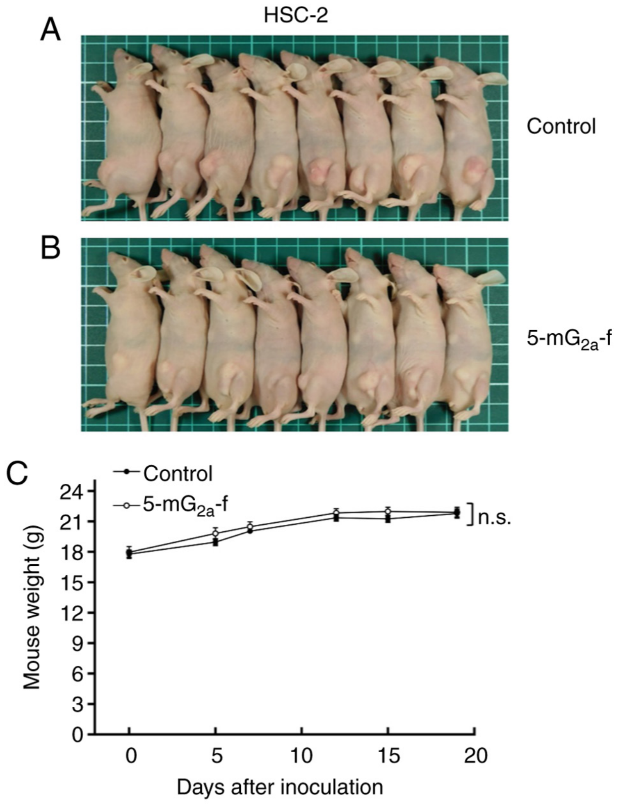

Figure S6. Body weights of mice implanted with HSC-2 xenografts and injected with 5-mG_{2a}-f after day 7. HSC-2 cells ($5x10^6$ cells) were injected subcutaneously into the left flank. After day 7, 100 μg of 5-mG_{2a}-f and control mouse IgG in 100 μl PBS were injected i.p. into treated and control mice, respectively. Additional antibodies were then injected on days 14 and 21. (A) Body appearance of mice with the HSC-2 xenografts of the control group on day 27. (B) Body appearance of mice with the HSC-2 xenografts of the 5-mG_{2a}-f group on day 27. (C) Body weights of the mice with the HSC-2 xenografts of 5-mG_{2a}-f group and control group were recorded on days 7, 12, 15, 19, 22, 26, and 27. Values are mean ± SEM. n.s., not significant (ANOVA and Sidak's multiple comparisons test).

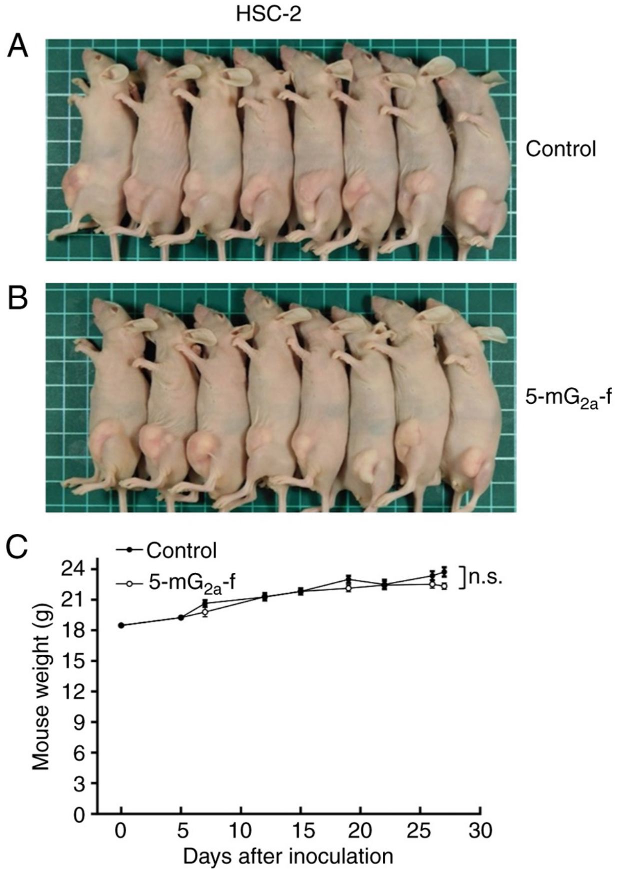

Supplement: Supporting Data [file Supplementary_Data.pdf]
